# Draft Genome Sequence of Calonectria pteridis, the Causal Agent of Calonectria Leaf Blight on Eucalyptus

**DOI:** 10.1128/mra.00284-22

**Published:** 2022-08-16

**Authors:** Marcia F. Queiroz, Samuel A. Santos, Pedro M. P. Vidigal, Túlio Morgan, Fernando M. Fernandes, Rafael F. Alfenas, Talyta G. Zarpelon, Acelino C. Alfenas

**Affiliations:** a Laboratory of Forest Pathology, Department of Plant Pathology, Instituto de Biotecnologia Aplicada à Agropecuária, Universidade Federal de Viçosa, Viçosa, Minas Gerais, Brazil; b Núcleo de Análise de Biomoléculas, Centro de Ciências Biológicas, Universidade Federal de Viçosa, Viçosa, Minas Gerais, Brazil; c Department of Microbiology, Instituto de Biotecnologia Aplicada à Agropecuária, Universidade Federal de Viçosa, Viçosa, Minas Gerais, Brazil; d Suzano Papel e Celulose, Aracruz, Espírito Santo, Brazil; University of Maryland School of Medicine

## Abstract

Here, we report the draft genome sequence of Calonectria pteridis, the causal agent of Calonectria leaf blight in eucalyptus plantations in Brazil. The 58,373,473-bp genome assembly consists of 1,167 scaffolds, with a GC content of 50.21%. These genomic data can contribute to future studies involving the biology, adaptability, and pathogenicity of C. pteridis.

## ANNOUNCEMENT

Calonectria pteridis (Cylindrocladium pteridis; Ascomycota, Sordariomycetes) is the causal agent of Calonectria leaf blight (CLB), a devastating foliar disease affecting eucalyptus plantations in Brazil (1, 2). Here, we present the characterization of the genome of C. pteridis LPF059, which was originally isolated from eucalyptus tree leaves with typical symptoms of CLB in Pará State, Brazil, and was deposited in the Culture Collection of the Laboratory of Forest Pathology (LPF), Universidade Federal de Viçosa (Viçosa, Brazil) (3). Mycelium grown on potato dextrose broth medium for 48 h at 26°C and 180 rpm was used for DNA extraction with the DNeasy Plant minikit (Qiagen) according to the manufacturer’s instructions. DNA quality and quantity were assessed through 1% agarose gel electrophoresis and use of a Qubit 2.0 fluorometer. A DNA library (insert size, ~350 bp) was prepared using the NEBNext Ultra II DNA library preparation kit (New England Biolabs, USA) following a standard protocol and was sequenced using the Illumina NovaSeq 6000 platform. Raw data (26,416,738 reads [2 × 150-bp paired-end reads]) were processed with FastQC v. 0.11.5 (4). Adapters and low-quality reads were trimmed with Trimmomatic v. 0.39 (Phred quality scores of ≥30) (5).

Trimmed reads (25,499,888 paired-end reads) were used to estimate the genome size by k-mer frequency and distribution using Jellifish v. 2.2.6 (6) to produce histograms of 17-, 21-, and 25-mers, which were processed by GenomeScope (7). The *ab initio* assembly of LPF059 genome was performed using SPAdes v. 3.12.0 (8), by testing different odd k-mer values (from 21 to 125). Scaffolding and gap-closing steps for the assembled contigs were performed by Redundans v. 0.14a with default parameters (9). To remove potential contaminants and mitochondrial sequences, all assembled scaffolds were compared with a bacterium-virus database and the mitochondrial genome of Calonectria ilicicola (GenBank accession number NC_046826.1) using BLASTn v. 2.6.0 (E values of 1e^−25^) (10). The completeness of the genome assembly was evaluated using BUSCO v. 5.0.0 (11) with the ascomycota_odb10 database.

The estimated genome size of C. pteridis LPF059 ranged from 56,565,321 to 56,655,434 bp. The genome assembly contains 1,167 scaffolds, with a total size of 58,373,473 bp, an *N*_50_ value of 322,939 bp, and a GC content of 50.21%. The longest scaffold was 1,316,024 bp. BUSCO completeness analysis showed 97.7% completeness (96.8% as single-copy genes and 0.9% as duplicated genes).

This work provided a basis for future studies on genetic population structure, comparative genomics, and transcriptomics, which may lead to a better understanding of the biology, adaptability, and pathogenesis of C. pteridis.

### Data availability.

The assembled genome sequence of C. pteridis isolate LPF059 was deposited in NCBI GenBank under accession number JAKZGU000000000. Raw data are available at the SRA under accession numbers SRR20046038 and SRR20046039. The BioProject and BioSample accession numbers are PRJNA812993 and SAMN26443383, respectively.
